# Percutaneous Removal of a Tricuspid Valve Vegetation Using the AngioVac System in a Young Woman Who Injects Drugs

**DOI:** 10.3390/diagnostics16193248

**Published:** 2026-10-08

**Authors:** Tomasz Skowerski, Andrzej Kułach, Marek Grabka, Piotr Pysz, Paulina Kudzia, Eliza Adamczyk-Filipek, Radosław Gocoł, Grzegorz Smolka

**Affiliations:** 1 Department of Cardiology, School of Health Sciences, Medical University of Silesia, 40-6340-6355 Katowice, Poland; 2 First Department of Cardiology, School of Medicine in Katowice, Medical University of Silesia, 40-6340-6355 Katowice, Poland; 3 Division of Cardiology and Structural Heart Diseases, School of Medicine in Katowice, Medical University of Silesia, 40-6340-6355 Katowice, Poland; 4 Department of Cardiac Surgery, School of Medicine in Katowice, Medical University of Silesia, 40-6340-6355 Katowice, Poland

**Keywords:** infective endocarditis, tricuspid valve, percutaneous mechanical aspiration, AngioVac, septic pulmonary embolism, people who inject drugs, transesophageal echocardiography, tricuspid regurgitation

## Abstract

Right-sided infective endocarditis (RSIE) is a difficult clinical problem, particularly among people who inject drugs (PWID), in whom large tricuspid vegetations may be complicated by severe regurgitation and septic pulmonary embolization. We describe a woman in her twenties with tricuspid valve infective endocarditis and bilateral cavitary pulmonary lesions consistent with septic emboli, in whom the vegetation enlarged from 18 × 11 mm to 18 × 20 mm and tricuspid regurgitation (TR) progressed from moderate to severe (vena contracta 12 mm, effective regurgitant orifice area 1.5 cm^2^, regurgitant volume 110 mL) over ten days of empirical anti-staphylococcal therapy. Surgery was considered by an individualized Heart Team assessment of the complete clinical course, but the patient declined open-heart surgery. After multidisciplinary Heart Team discussion, transesophageal echocardiography (TEE)-guided percutaneous aspiration with the AngioVac system was undertaken as an individualized debulking and source-control strategy, not as an equivalent alternative to surgery. Under general anesthesia, bilateral femoral venous access was obtained and seven aspiration passes retrieved four large vegetation fragments, with no residual intracardiac mass on completion TEE. Culture of the aspirated material yielded *Serratia marcescens* and *Escherichia coli*, neither of which was covered by the empirical regimen, which was replaced by susceptibility-guided ciprofloxacin and co-trimoxazole; ceftazidime was added when *Pseudomonas aeruginosa* was later isolated from blood. Residual regurgitation arose from a flail, prolapsing posterior leaflet rather than from mass effect—a finding that may reflect pre-existing infective leaflet destruction, leaflet injury during aspiration, or both—and was quantitatively reduced three to four weeks after the procedure (vena contracta 6 mm, effective regurgitant orifice area 0.55 cm^2^, regurgitant volume 36 mL); at six months it was moderate-to-severe and stable, with preserved right ventricular function (TAPSE 30 mm) and no recurrent vegetation. This case supports a potential diagnostic and therapeutic role for AngioVac-assisted debulking as an individualized adjunct in highly selected patients with RSIE who refuse surgery, with the caveat that aspiration reduces vegetation and embolic burden without correcting the underlying valvular lesion.

Right-sided infective endocarditis (RSIE) accounts for 5–10% of all cases of infective endocarditis and is frequently associated with injection drug use [1]. Percutaneous mechanical aspiration (PMA) has emerged over the past decade as an option for debulking large right-sided vegetations in patients in whom surgery is indicated but not feasible or not accepted [2,3,4]. We present a case of percutaneous removal of a tricuspid valve vegetation using the AngioVac system (AngioDynamics, Latham, NY, USA).

A woman in her twenties with opioid use disorder (injected oxycodone), bipolar disorder, previous Mycoplasma pneumoniae infection, previous methicillin-sensitive Staphylococcus aureus sepsis, acute kidney injury, and heart failure (New York Heart Association class II) was admitted with tricuspid valve infective endocarditis. On admission she was hemodynamically stable (heart rate 96/min; blood pressure 123/80 mmHg; oxygen saturation 97%; temperature 36.8 °C). Laboratory tests showed anemia (hemoglobin 8.3 g/dL) and elevated inflammatory markers (C-reactive protein 126 mg/L; neutrophils 10.34 × 10^3^/µL; procalcitonin 0.15 ng/mL). Blood cultures obtained on admission remained negative. High-resolution chest computed tomography revealed bilateral thick-walled cavitary lesions with air–fluid levels consistent with septic pulmonary emboli, a right-sided pleural effusion of up to 33 mm, cardiomegaly, splenomegaly, and ascites.

Serial echocardiography documented rapid progression during empirical antimicrobial therapy (Table S1). On the initial transesophageal study the tricuspid leaflets were thin and mobile, a mobile oval mass of 18 × 11 mm was attached to the posterior leaflet at its junction with the annulus, and tricuspid regurgitation (TR) was moderate. Three days later, the mass measured 16 × 15 mm and TR had become severe (effective regurgitant orifice area [EROA] 1.0 cm^2^, regurgitant volume 94 mL, TR Vmax 2.7 m/s, estimated right ventricular systolic pressure 33 mmHg). On the pre-procedural study, the mass measured 18 × 20 mm in the four-chamber view and 17 × 14 mm in the right ventricular inflow view and prolapsed between the right ventricle and the right atrium. TR was severe by an integrative multiparametric assessment [5]: vena contracta 12 mm, EROA 1.5 cm^2^, regurgitant volume 110 mL, intermittent systolic reversal of hepatic venous flow, dilated inferior vena cava (20 mm, collapsibility >50%), tricuspid annular diameter 32–34 mm and right atrial area 21 cm^2^ (volume 72 mL). The right ventricle was dilated (basal diameter 46 mm) with preserved longitudinal function (TAPSE 33 mm), and left ventricular function was preserved throughout.

The case was discussed by a multidisciplinary Heart Team. In the 2023 ESC guidelines, surgical treatment of right-sided endocarditis is reserved for patients, on appropriate antimicrobial therapy, with right ventricular dysfunction secondary to acute severe tricuspid regurgitation, persistent vegetations with respiratory insufficiency requiring ventilatory support after recurrent pulmonary emboli, large residual tricuspid vegetations (>20 mm) after recurrent septic pulmonary emboli, or simultaneous involvement of left-sided structures, and for persistent bacteremia despite appropriate antimicrobial therapy [1]; an isolated vegetation, whatever its size, is not in itself an indication for surgery. None of these criteria was fulfilled by a single finding in this patient. The Heart Team’s conclusion that surgery was indicated was an individualized assessment of the complete clinical course rather than the application of a size threshold: a vegetation that enlarged from 11 to 20 mm within ten days of antimicrobial therapy, established bilateral cavitary septic pulmonary emboli, and deterioration from moderate to severe regurgitation with right ventricular dilatation during the first ten days of admission. Both valve repair and replacement were considered. Ongoing injection drug use is not in itself a contraindication to otherwise indicated cardiac surgery, and current statements caution against withholding surgery on this basis alone [6]; the anticipated risk of prosthetic reinfection was therefore weighed as one element of shared decision-making rather than treated as an exclusion criterion. The decisive factor in this case was the patient’s informed and repeatedly confirmed refusal of open-heart surgery. Against this background, TEE-guided percutaneous aspiration was proposed as an individualized debulking and source-control strategy, with the aim of reducing vegetation and embolic burden and obtaining tissue for microbiological diagnosis, and with the understanding that it would not address the underlying valvular lesion.

The procedure was performed under general anesthesia with continuous TEE guidance via bilateral femoral venous access (left femoral vein reinfusion cannula; right femoral vein, AngioVac inflow cannula) after pre-closure with two suture-mediated closure devices. Systemic heparin was administered. Seven aspiration passes were performed. Four large vegetation fragments were retrieved (Figure 1D). There were no vascular access complications, air embolism, arrhythmia or hemodynamic instability, and the patient was extubated in the catheterization laboratory.

Completion TEE showed no residual intracardiac mass, persistent significant tricuspid regurgitation and a flail posterior leaflet. The pre-procedural studies had documented thin, mobile leaflets with a prolapsing mass but no flail segment. Because a flail segment was not unequivocally documented before aspiration, it is not possible to determine whether the flail posterior leaflet seen on completion TEE represents pre-existing infective leaflet destruction unmasked by removal of the vegetation, leaflet injury sustained during aspiration, or a combination of the two; the sequence of findings does not establish causality in either direction, and procedural leaflet injury—a recognized complication of percutaneous aspiration [2,3,4]—cannot be excluded. Quantitative reassessment approximately three to four weeks after the procedure nevertheless showed a substantial reduction in regurgitant severity compared with the pre-procedural study: vena contracta 6 versus 12 mm, EROA 0.55 versus 1.5 cm^2^, regurgitant volume 36 versus 110 mL, TR Vmax 2.1 versus 2.7 m/s, and right ventricular basal diameter 41 versus 46 mm (Table S1). The residual regurgitation was eccentric and arose from leaflet prolapse, with 5 mm thickening of the anterior leaflet and no residual vegetation; it was graded severe on one study and moderate-to-severe four days later, its quantitative parameters lying at the boundary between the two grades. We interpret this sequence as removal of the mass effect of a large mobile vegetation that had prevented leaflet coaptation, superimposed on structural leaflet damage—infective, procedural or both—that aspiration cannot repair: regurgitation improved in degree but not in mechanism. Two qualifications apply. The therapeutic target of the intervention was reduction of vegetation and embolic burden, not restoration of valve competence, and no quantitative TR assessment was obtained on the immediate post-procedural study, so the early hemodynamic effect of aspiration cannot be separated from the subsequent resolution of the acute septic state.

The microbiological findings require explanation. Two sets of blood cultures obtained on admission were negative. Empirical therapy with cefazolin 2 g every 8 h and cloxacillin 3 g every 6 h was directed at staphylococci in view of the patient’s previous methicillin-sensitive S. aureus sepsis. Culture of the aspirated vegetation grew *Serratia marcescens* and *Escherichia coli*. Neither organism was covered by that regimen, since *S. marcescens* is intrinsically resistant to first-generation cephalosporins and cloxacillin has no Gram-negative activity; the enlargement of the vegetation and the progression of regurgitation documented over the preceding ten days therefore took place during therapy that, in retrospect, was inactive against the causative organisms. Both agents were stopped when the result became available and replaced with susceptibility-guided ciprofloxacin 400 mg and co-trimoxazole 960 mg, each every 12 h intravenously. Aspiration thus provided the only microbiological diagnosis in this patient and changed antimicrobial therapy completely.

Five days after the procedure, blood cultures grew *Pseudomonas aeruginosa*, and ceftazidime 2 g every 8 h was added, giving dual anti-pseudomonal cover with ciprofloxacin. We interpreted this isolate as a new healthcare-associated bloodstream infection rather than a third endocardial pathogen, given its timing late in a prolonged admission, the presence of an indwelling venous catheter, and the absence of recurrent vegetation, new septic emboli or clinical deterioration on concurrent imaging. New bloodstream infections complicate approximately one in five episodes of infective endocarditis among people who inject drugs, are predominantly caused by Gram-negative bacilli, and are frequently polymicrobial [7]. After three weeks of intravenous organism-directed therapy, ciprofloxacin and co-trimoxazole were switched to the oral route (500 mg and 1920 mg twice daily, respectively) three days before discharge and continued to a planned total duration of four weeks; ceftazidime was continued intravenously for two weeks, until discharge. The four-week course was counted from the first day of effective, susceptibility-guided therapy, which began after source control had been achieved by aspiration of the vegetation, and not from the procedure itself or from a documented bloodstream clearance, because blood cultures for the endocardial pathogens were negative throughout. The duration was chosen [in consultation with an infectious diseases specialist] on the basis of complete removal of the infected material with no residual mass on imaging, negative blood cultures before and after the procedure, and a prompt clinical and biochemical response. We acknowledge that four weeks is shorter than the six weeks or more generally advised for non-HACEK Gram-negative endocarditis [1], a recommendation that rests on limited observational data [8,9]; the patient was therefore followed closely, and no clinical, microbiological or echocardiographic relapse had occurred at six months.

Contamination of the aspirate cannot be excluded on principle, because material retrieved with the AngioVac system passes through an extracorporeal circuit and a filter canister and is not a sterile-site specimen in the way that a surgically excised valve is. Several observations argue against it: the clinical and biochemical response to organism-directed therapy was prompt, and both isolates are plausible in this setting. Serratia spp. endocarditis has emerged as a distinct entity among people who inject drugs, with a rising incidence reported across several centers [8]; non-HACEK Gram-negative organisms account for a small but growing proportion of cases [9], and the combination of a water-associated environmental organism with an enteric one is consistent with contaminated injection equipment. *S. marcescens* and *P. aeruginosa* are listed as typical organisms in the 2023 Duke–ISCVID criteria only in the presence of intracardiac prosthetic material [10]; on a native valve, the diagnosis in this patient rested on culture of the vegetation itself rather than on the species isolated. Evidence to guide treatment of non-HACEK Gram-negative endocarditis remains limited and largely observational [8,9].

During hospitalization, the cavitary pulmonary lesions partially regressed, inflammatory markers decreased and repeat imaging showed no residual or recurrent vegetation. Thoracic surgical consultation did not indicate surgical management of the pulmonary lesions. Blood cultures were negative at discharge. At six months, transthoracic echocardiography showed no vegetation, preserved left ventricular function (LVEF 55%), preserved right ventricular longitudinal function (TAPSE 30 mm), a right ventricular basal diameter of 44 mm, and moderate-to-severe tricuspid regurgitation (vena contracta 6 mm, TR Vmax 2.5 m/s, peak gradient 25 mmHg), unchanged from early post-procedural studies (Table S1).

This case shows both of the contributions that percutaneous aspiration can make: mechanical debulking of a large, rapidly enlarging vegetation with high embolic potential, and retrieval of tissue that established the microbiological diagnosis and redirected therapy in a patient with negative blood cultures. It shows the limitations just as clearly. Aspiration does not repair the structurally damaged valve, may itself injure leaflet tissue, does not reliably eradicate organisms embedded within leaflet or annular tissue, and does not remove the risk of recurrence, particularly where injection drug use continues; moderate-to-severe regurgitation persisted at six months and the patient remains a candidate for later definitive valve surgery. The evidence base is retrospective and short-term. In the largest series to date, procedural success was 89% but the composite of in-hospital death, new pulmonary embolism or emergency surgery occurred in 18% [2], and no validated patient-selection criteria exist [2,3,4,11,12]. Data from series of heterogeneous right-heart masses, such as the RAPID registry [13], should not be extrapolated to infected vegetations. Percutaneous aspiration is therefore best regarded as an individualized adjunct in highly selected patients, including as a bridge to later definitive surgery, and not as an established equivalent to tricuspid valve surgery in RSIE.

## Figures and Tables

**Figure 1:**
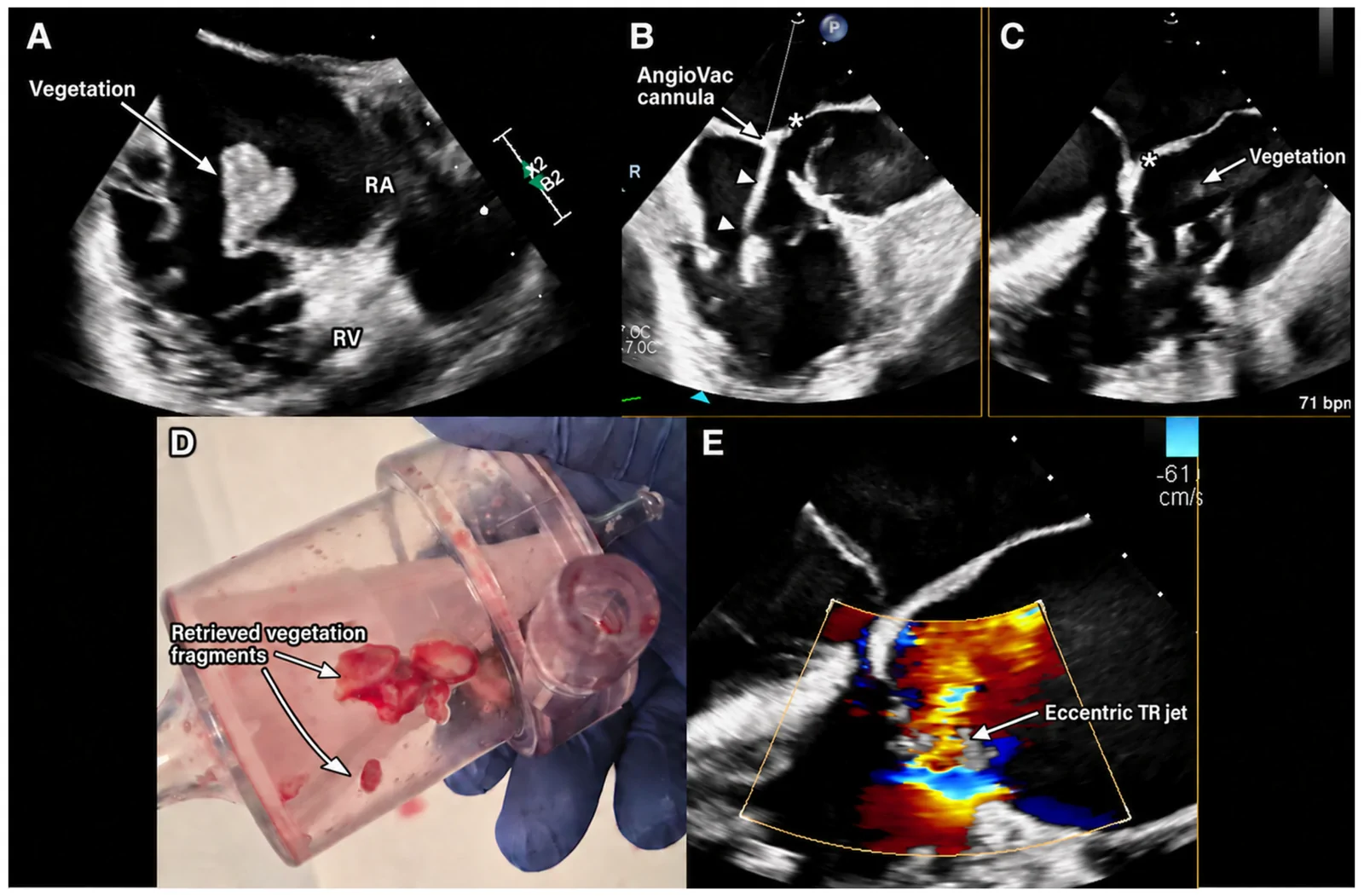
Percutaneous aspiration of a tricuspid valve vegetation with the AngioVac system. (**A**) Baseline mid-esophageal transesophageal echocardiographic (TEE) view: a mobile, lobulated vegetation (arrow) attached to the posterior tricuspid leaflet; RA, right atrium; RV, right ventricle; the calipers indicate the maximal diameter of the vegetation. (**B**) The AngioVac inflow cannula (arrowheads) advanced across the right atrium towards the tricuspid annulus under TEE guidance; the asterisk marks the cannula tip. (**C**) Aspiration: the cannula tip (asterisk) engages the vegetation (arrow), which is drawn into the cannula. (**D**) The four retrieved vegetation fragments in the AngioVac filter canister. (**E**) Post-procedural color Doppler TEE showing a persistent broad, eccentric tricuspid regurgitant jet; quantitative assessment before and after the procedure is given in Table S1.

## Data Availability

The data supporting the findings of this report are contained within the article. Further anonymized data are available from the corresponding author upon reasonable request.

## Supplementary Materials

The following supporting information can be downloaded at: https://www.mdpi.com/article/10.3390/diagnostics16193248/s1, Table S1: Serial echocardiographic assessment before and after percutaneous aspiration. EROA, effective regurgitant orifice area; LVEF, left ventricular ejection fraction; PISA, proximal isovelocity surface area; RA, right atrium; RV, right ventricle; RVSP, right ventricular systolic pressure; TAPSE, tricuspid annular plane systolic excursion; TEE, transesophageal echocardiography; TR, tricuspid regurgitation; TTE, transthoracic echocardiography.
