# Supplementary material for: Percutaneous Removal of a Tricuspid Valve Vegetation Using the AngioVac System in a Young Woman Who Injects Drugs
Source: Diagnostics (Basel). 2026 Oct 8;16(19):3248. doi: 10.3390/diagnostics16193248 (PMC13664219; doi:10.3390/diagnostics16193248)
Supplement: Supplementary file 1 [file diagnostics-16-03248-s001.zip › diagnostics-4487307-supplementary.pdf]

Supplement materials

Table S1. Serial echocardiographic assessment before and after percutaneous aspiration. EROA, effective regurgitant orifice area; LVEF, left ventricular ejection fraction; PISA, proximal isovelocity surface area; RA, right atrium; RV, right ventricle; RVSP, right ventricular systolic pressure; TAPSE, tricuspid annular plane systolic excursion; TEE, transesophageal echocardiography; TR, tricuspid regurgitation; TTE, transthoracic echocardiography.

Parameter	TEE, Day −14	TTE, Days −11 to −4 (Pre-Procedure)	TTE, Days +24 to +28 (Post-Procedure)	TTE, 6 Months (Day +188)
Vegetation, maximal dimensions	18 × 11 mm	16 × 19 mm and 16 × 15 mm (day −11, two studies); 18 × 20 mm (day −4)	None	None
Leaflet morphology	Thin, mobile leaflets; mass at annular margin	Mass prolapsing between RV and RA	Leaflet prolapse; anterior leaflet thickened to 5 mm	No vegetation
TR grade	Moderate (II)	Severe (III)	Severe (day +24); moderate-to-severe (day +28)	Moderate-to-severe
TR jet	Not described	Not described	Eccentric	Not described
Vena contracta width	Not reported	12 mm	6 mm	6 mm
EROA (PISA)	Not reported	1.5 cm² (1.0 cm² on day −11)	0.55 cm²	Not reported
Regurgitant volume	Not reported	110 mL (94 mL on day −11)	36 mL	Not reported
TR Vmax	Not reported	2.7 m/s (day −11)	2.1 m/s	2.5 m/s
Estimated RVSP/peak TR gradient	Not reported	RVSP 33 mmHg (day −11)	Peak gradient 17 mmHg	Peak gradient 25 mmHg
Systolic hepatic vein flow reversal	Not reported	Present, intermittent	Not reported	Not reported
Inferior vena cava	Not reported	20 mm, collapsibility >50%	Not reported	Not reported
Tricuspid annular diameter	Not reported	32–34 mm	Not reported	Not reported
RV basal diameter	35 mm	46 mm (day −11)	41–42 mm	44 mm
TAPSE	Not reported	33 mm	27 mm	30 mm
RA area / volume	Not reported	21 cm²/72 mL	21 cm²/not reported	Not reported
LVEF	Not reported	55–60%	65% (day +24); 55% (day +28)	55%

Time points are given in days relative to the procedure (day 0). Values are given as documented in the source reports; where two studies were performed on the same day, both values are shown.
